# Perineural Invasion Is a Significant Indicator of High Malignant Degree and Poor Prognosis in Esophageal Cancer: A Systematic Review and Meta-Analysis

**DOI:** 10.3389/fonc.2022.816270

**Published:** 2022-06-08

**Authors:** Liuyang Bai, Liangying Yan, Yaping Guo, Luyun He, Zhiyan Sun, Wenbo Cao, Jing Lu, Saijun Mo

**Affiliations:** ^1^Department of Pathophysiology, School of Basic Medical Sciences, Zhengzhou University, Zhengzhou, China; ^2^Collaborative Innovation Center of Henan Province for Cancer Chemoprevention, Zhengzhou, China; ^3^State Key Laboratory of Esophageal Cancer Prevention and Treatment, Zhengzhou, China; ^4^Department of Special Service, No. 988 Hospital of the Joint Service Support Force of People’s Liberation Army of China (PLA), Zhengzhou, China

**Keywords:** esophageal cancer, perineural invasion, lymphovascular invasion, lymph node metastasis, prognosis

## Abstract

**Background:**

Perineural invasion (PNI) is a malignant metastatic mode of tumors and has been reported in many tumors including esophageal cancer (EC). However, the role of PNI in EC has been reported differently. This systematic review and meta-analysis aims to focus on the role of PNI in EC.

**Methods:**

Eight databases of CNKI, VIP, Wanfang, Scopus, Wiley, ISI, PubMed, and EBSCO are used for literature search. The association of PNI with gender, pathological stages of T and N (pT and pN), lymphovascular invasion (LVI), lymph node metastasis, 5-year overall survival (OS), and 5-year disease-free survival (DFS) was examined in the meta-analysis by Revman5.0 Software. The pooled OR/HR and 95% CI were used to assess the risk and prognostic value.

**Results:**

Sixty-nine published studies were screened for analysis of PNI in EC. The incidence of PNI in esophageal squamous carcinoma (ESCC) and esophageal adenocarcinoma (EAC) was different, but not statistically significant (*p >* 0.05). The PNI-positive patients had a significantly higher risk of pT stage (OR = 3.85, 95% CI = 2.45–6.05, *p* < 0.00001), pN stage (OR = 1.86, 95% CI = 1.52–2.28, *p* < 0.00001), LVI (OR = 2.44, 95% CI = 1.55–3.85, *p* = 0.0001), and lymph node metastasis (OR = 2.87, 95% CI = 1.56–5.29, *p* = 0.0007). Furthermore, the cumulative analysis revealed a significant correlation between PNI and poor OS (HR = 1.37, 95% CI = 1.24–1.51, *p* < 0.0001), as well as poor DFS (HR = 1.55, 95% CI = 1.38–1.74, *p* < 0.0001).

**Conclusion:**

PNI occurrence is significantly related to tumor stage, LVI, lymph node metastasis, OS, and DFS. These results indicate that PNI can serve as an indicator of high malignant degree and poor prognosis in EC.

## Introduction

Esophageal cancer (EC) is one of the top ten malignant tumors. According to global cancer statistics in 2020, EC ranks seventh in terms of incidence and sixth in mortality overall (1). The histological types of EC mainly contain esophageal squamous cell carcinoma (ESCC) and esophageal adenocarcinoma (EAC). ESCC is the most common histological type in China, accounting for more than 90%. In EC treatment, surgical excision is the best treatment for the early stage, and radiotherapy and chemotherapy are often used for the middle and late stage. Recently, neoadjuvant chemoradiation (nCRT) followed by esophagectomy is increasingly applied to locally advanced EC. However, the prognosis of EC is still poor after these treatments due to its insidious and highly invasive nature in the early stage. Most EC patients are prone to relapse and metastasis. In recent years, researchers found that there is a new metastasis pathway, perineural invasion (PNI), often happening in EC patients.

PNI refers to the phenomenon of cancer cells surrounding nerve fibers and entering the surrounding nerve, spreading local infiltration and metastasis. Now, the definition of PNI is that the tumor cells are in close contact with the nerve and surround at least 33% of the nerve periphery or invade any of the three layers of the nerve sheath, which is also taken as the current pathological diagnostic criteria for PNI (2, 3). The occurrence of PNI not only is with incomplete resection of the tumor and recurrence of prognosis, but also often leads to pain in many cancers, such as prostate cancer, pancreatic cancer, and head and neck cancer (2–5). However, the role of PNI in EC is differently reported. For example, PNI is associated with poor overall survival (OS) and can serve as an independent factor for OS in multivariate analysis (6–8), while Lee et al. thought PNI was not an important prognostic parameter in EC (9). These inconsistent conclusions may be due to the insufficient sample size. Hence, we collected a larger number of data from EC patients and used a systematic review and meta-analysis to obtain more accurate conclusions of PNI. The study determined the association of PNI with pathological parameters, OS and DFS, and then evaluated the role and effect of PNI on EC.

## Materials and Methods

### Literature Search

A literature search was performed by using the CNKI, VIP, Wanfang, Scopus, Wiley, ISI, PubMed, and EBSCO databases from January 1, 1990 to March 30, 2022. The main keywords in the abstract were “perineural invasion”, “esophageal”, and “cancer”. The articles in CNKI, VIP, and Wanfang databases only come from the Chinese Core Journal. Duplicate articles were deleted and full articles were used for analysis. The articles with patient samples from the same institution in the repeated recruitment period, reviews, and case reports were excluded. The literatures of esophageal neuroendocrine carcinoma were also excluded. The quality of all studies was assessed by using the Newcastle–Ottawa Scale and was scored from 6 to 8 (full score = 9).

### Literature Extraction

To analyze the positive rate of PNI in different pathological types of EC, the following information in the articles was extracted: first author, year of publication, country of study, patient samples of recruitment period, pathological types of EC (ESCC and EAC), and the number of samples who are PNI positive.

For meta-analysis to examine the association of PNI with gender, pT stage, pN stage, lymphovascular invasion (LVI), lymph node metastasis, 5-year OS, and 5-year disease-free survival (DFS), the extracted information contains the following: first author, year of publication, odds ratio (OR), hazard ratio (HR), and the corresponding 95% confidence interval (CI). The pooled HR and 95% CI were calculated using the method of inverse variance and the *p*-value threshold was set at 0.05. Some articles do not directly provide HR data, but provide RR data. HR and RR can be combined because without considering the time factor in the paper, they represent the same meaning.

### Statistical Analysis

Statistical calculation was completed with SPSS21.0 software and statistical heterogeneity was tested using the Chi-square test. *p* < 0.05 indicated statistical significance. The forest and funnel plots of the meta-analysis were made using Review Manager 5.0 software (Revman5.0). *p* < 0.10 or/and *I*² > 50% were used to indicate heterogeneity.

## Results

### Literature Search Results

**Figure 1:**
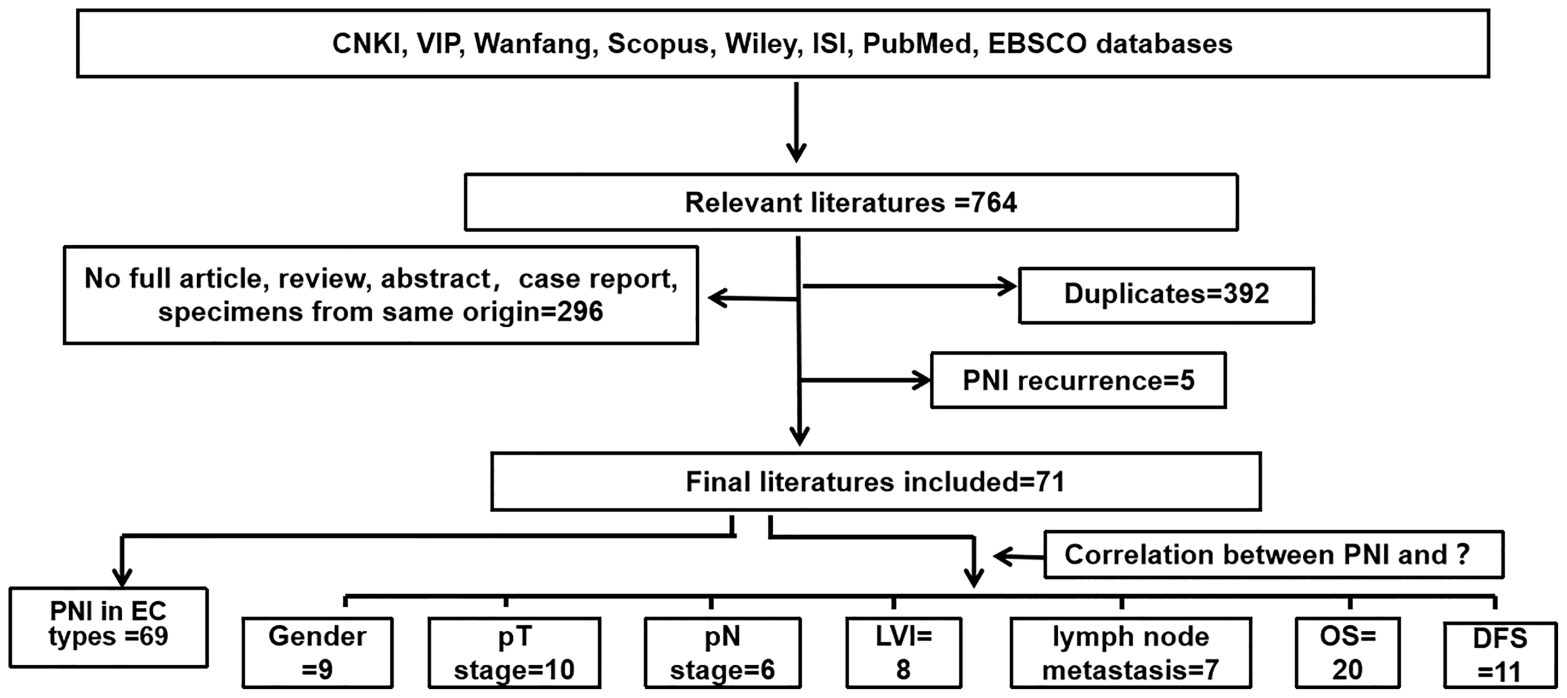
Flow chart of the database search.

The systematic search identified 764 potentially eligible articles, 392 of which were excluded due to duplication. Of the remaining 372 studies, 296 were excluded because they were reviews or specimens of the same origin. Five studies were excluded for PNI recurrence after neoadjuvant therapy or chemotherapy. Finally, 71 studies were finally included in this study: 69 studies were used to analyze the positive rate of PNI in different pathological types of EC; 9, 10, 6, 8, 7, 20, and 11 studies were used to analyze the correlation between PNI and gender, pT stage, pN stage, LVI, lymph node metastasis, OS, and DFS, respectively. The detailed screening process is shown in **Figure 1**.

### The PNI Occurrence Is Different Between ESCC and EAC

A total of 69 studies were used to analyze the distribution of PNI in different pathological types of EC, including 24 studies on EC (ESCC, EAC, and other types including esophageal small cell undifferentiated carcinoma and esophagus carcinosarcoma) (6–8, 10–30), 32 studies on ESCC (9, 31–61), and 13 studies on EAC (62–74). The detailed information is shown in **Table 1**. The median of PNI incidence of EC, ESCC, and EAC was 33% (range from 5% to 66%), 24% (range from 6% to 85%), and 46% (range from 20% to 56%), respectively. Data analysis showed that the PNI occurrence rate has no significant difference between ESCC and EAC (**Figure S1A**).

**Table 1:** The distribution of PNI in different pathological types of esophageal cancer.

Study	Country	Re. period	N	PNI+(%)	Male(%)	T stage(T1+T2/T3+T4)	N stage(N-/N+)	LVI+()	LNM+()	Outcome	Study quality
**EC**											
**Alcan S, 2022 (10)**	**Turkey**	**2008-2018**	**50**	**30(60%)**	**33(66%)**	**13/37**	**21/29**	**-**	**-**	**OS**	**7**
**Yıldırım ÖA, 2022 (11)**	**Turkey**	**2011-2021**	**64**	**10(15.6%)**	**44(69%)**	**-**	**-**	**15(23%)**	**-**	**-**	**7**
**Huang Z, 2022 (12)**	**China**	**2019-2020**	**533**	**180(33.8%)**	**433(81.2%)**	**201/332**	**247/286**	**326(61.2%)**	**286(53.7%)**	**-**	**8**
**Wang YP, 2020 (6)**	**China**	**2012-2016**	**162**	**32(19.8%)**	**127(79.5%)**	**66/96**	**82/80**	**-**	**-**	**OS DFS**	**7**
**Zheng CY, 2019 (13)**	**China**	**2014-2016**	**182**	**120(65.9%)**	**144(79.1%)**	**112/70**	**-**	**-**	**-**	**-**	**6**
**Velickovic D, 2019 (14)**	**Serbia**	**2004-2016**	**409**	**135(33%)**	**333(81.4%)**	**86/316**	**80/325**	**-**	**-**	**-**	**7**
**Zhang WY, 2018 (15)**	**China**	**2006-2012**	**408**	**72(17.6%)**	**357(87.5%)**	**173/235**	**205/203**	**-**	**-**	**-**	**7**
**Zeng YZ, 2018 (16)**	**China**	**2014-2016**	**141**	**30(21.3%)**	**56(39.7%)**	**10/61**	**-**	**-**	**-**	**-**	**6**
**Miao N, 2018 (17)**	**China**	**2007-2014**	**250**	**47(18.8%)**	**175(70%)**	**115/115**	**165/85**	**-**	**-**	**-**	**7**
**Faiz Z, 2018 (18)**	**Netherlands**	**2000-2015**	**81**	**51(63%)**	**-**	**0/81**	**22/59**	**-**	**-**	**OS DFS**	**7**
**Zhu TY, 2017 (19)**	**China**	**2012-2013**	**177**	**116(65.5%)**	**150(84.7%)**	**108/69**	**-**	**-**	**111(62.7%)**	**-**	**7**
**Gao X, 2017 (20)**	**China**	**2008-2012**	**247**	**92(37.2%)**	**149(60.3%)**	**-**	**-**	**-**	**128(51.8%)**	**-**	**6**
**Li ZY, 2016 (21)**	**China**	**2010-2015**	**1160**	**147(12.7%)**	**830(71.6%)**	**296/864**	**540/620**	**-**	**-**	**OS**	**7**
**Sun YH, 2015 (7)**	**China**	**1981-2011**	**26**	**5(19.2%)**	**23(88.5%)**	**10/16**	**16/10**	**7(26.9%)**	**-**	**OS DFS**	**7**
**Dong X, 2014 (22[]**	**China**	**2007-2010**	**248**	**14(6%)**	**184(74.2%)**	**114/134**	**-**	**127(51.2%)**	**-**	**-**	**7**
**Tachezy M, 2014 (23)**	**Germany**	**1992-2009**	**644**	**36(6%)**	**517(80.3%)**	**295/347**	**242/395**	**209(32.5%)**	**-**	**-**	**7**
**Dolan JP, 2013 (24)**	**USA**	**1995-2011**	**146**	**85(58.2%)**	**120(84.5%)**	**-**	**-**	**-**	**-**	**-**	**6**
**Noble F, 2013 (8)**	**UK**	**2005-2010**	**246**	**34(13.8%)**	**195(79.3%)**	**104/118**	**128/118**	**-**	**-**	**OS DFS**	**7**
**Gray RT, 2012 (25)**	**UK**	**1999-2000**	**42**	**15(35.7%)**	**35(83.3%)**	**13/29**	**28/14**	**36(85.7%)**	**-**	**-**	**7**
**Fassan M, 2010 (26)**	**Italy**	**2002-2006**	**111**	**37(33.3%)**	**93(83.8%)**	**-**	**-**	**-**	**-**	**-**	**6**
**Izzo JG, 2006 (27)**	**USA**	**NR**	**43**	**16(37.2)**	**41(95%)**	**5/38**	**11/32**	**-**	**-**	**-**	**7**
**Khan OA, 2004 (28)**	**UK**	**1987-2001**	**219**	**11(5%)**	**145(66.2%)**	**-**	**-**	**-**	**-**	**-**	**6**
**Glickman JN, 1999 (29)**	**USA**	**1985-1996**	**145**	**52(35.9%)**	**-**	**-**	**-**	**-**	**67(46.2%)**	**-**	**6**
**Tanaka A, 1998 (30)**	**Japan**	**NR**	**104**	**48(46.2%)**	**84(80.8%)**	**30/74**	**46/55**	**69(66.3%)**	**55(52.9%)**	**-**	**7**
**ESCC**										**-**	
**Cheng J, 2022 (31)**	**China**	**2021-2022**	**149**	**15(10%)**	**123(82.6%)**	**5/144**	**10/139**	**8(5%)**	**-**	**-**	**7**
**Xie C, 2022 (32)**	**China**	**2012-2018**	**195**	**42(21.5%)**	**140(71.8%)**	**72/122**	**91/104**	**-**	**91(46.7%)**	**-**	**7**
**Li A, 2021 (33)**	**China**	**2015-2020**	**143**	**39(27.3%)**	**85(59.4%)**	**-**	**-**	**-**	**-**	**-**	**6**
**Peng H, 2021 (34)**	**China**	**2013-2017**	**121**	**12(10%)**	**96(79.3%)**	**55/66**	**-**	**-**	**58(47.9%)**	**OS**	**7**
**Yeh JC, 2021 (35)**	**China**	**2009-2017**	**278**	**63(22.7%)**	**251(90.3%)**	**95/128**	**180/98**	**80(28.8%)**	**-**	**-**	**8**
**Zeng YZ, 2021 (36)**	**China**	**2014-2016**	**97**	**10(10.3%)**	**78(80.4%)**	**17/80**	**41/56**	**-**	**56(57.7%)**	**DFS**	**7**
**Li QM, 2020 (37)**	**China**	**2015-2019**	**443**	**58(13.1%)**	**259(58.5%)**	**277/166**	**-**	**-**	**117(26.4%)**	**-**	**7**
**Tian H, 2020 (38)**	**China**	**2016-2018**	**150**	**35(23.3%)**	**102(68%)**	**70/80**	**-**	**-**	**60(40%)**	**-**	**7**
**Cui J, 2020 (39)**	**China**	**2012-2018**	**407**	**210(51.6%)**	**390(95.8%）**	**114/293**	**-**	**-**	**232(57%)**	**-**	**7**
**Guo YN, 2020 (40)**	**China**	**2009-2013**	**162**	**119(73.5%)**	**108(66.7%)**	**30/162**	**-**	**-**	**-**	**-**	**6**
**Lee HK, 2020 (9)**	**Korea**	**2000-2018**	**64**	**13(20.3%)**	**60(93.8%)**	**36/28**	**39/25**	**-**	**-**	**OS DFS**	**7**
**Tang Y, 2019 (41)**	**China**	**2010-2015**	**347**	**44(12.7%)**	**267(76.9%)**	**-**	**-**	**-**	**-**	**-**	**6**
**Lin G, 2019 (42)**	**China**	**2011-2017**	**101**	**86(85.1%)**	**78(77.2%)**	**50/50**	**-**	**-**	**-**	**-**	**7**
**Rong L, 2019 (43)**	**China**	**1999-2003**	**378**	**125(33.1%)**	**307(81.2%)**	**103/275**	**189/189**	**-**	**-**	**OS DFS**	**7**
**Wang H, 2018 (44)**	**China**	**2008-2014**	**117**	**30(25.6%)**	**87(74.4%)**	**60/57**	**75/42**	**-**	**43 (36.8%)**	**-**	**7**
**Hong ZP, 2018 (45)**	**China**	**2014-2017**	**108**	**43(39.8%)**	**106(98.1%)**	**10/98**	**-**	**-**	**87(80.6%)**	**-**	**7**
**Tsai CY, 2017 (46)**	**China**	**1998-2008**	**177**	**77(43.5)**	**171 (96.6%)**	**60/117**	**103/74**	**71 (40.1%)**	**-**	**-**	**7**
**Tu CC, 2017 (47)**	**China**	**2009-2014**	**91**	**15(16.5%)**	**88(96.7%)**	**21/70**	**27/54**	**21(23.1%)**	**-**	**OS DFS**	**7**
**Wang H, 2017 (48)**	**China**	**2010-2015**	**446**	**113(25.3%)**	**310(69.5%)**	**70/376**	**282/164**	**-**	**164(36.8%)**	**OS**	**8**
**Xu G, 2017 (49)**	**China**	**2008-2011**	**302**	**153(50.7%)**	**233(77.2%)**	**146/156**	**165/137**	**-**	**-**	**DFS**	**7**
**Hsieh CC, 2016 (50)**	**China**	**2006-2013**	**81**	**24(29.6%)**	**70(86.4%)**	**24/57**	**31/50**	**36(44.4%)**	**-**	**OS DFS**	**8**
**Wu J, 2016 (51)**	**China**	**2003-2010**	**1435**	**274(19.1%)**	**1254(87.4%)**	**430/1005**	**671/764**	**-**	**-**	**OS**	**7**
**Sato-Kuwabara Y, 2016 (52)**	**Brazil**	**1980-1999**	**95**	**27(28.4%)**	**78(82.1%)**	**-**	**-**	**-**	**-**	**-**	**6**
**Ning ZH, 2015 (53)**	**China**	**2005-2010**	**243**	**54(22.2%)**	**194(79.8%)**	**51/192**	**106/137**	**-**	**-**	**OS DFS**	**7**
**Park SY, 2015 (54)**	**Korea**	**2010-2014**	**85**	**5(6%)**	**77(90.6%)**	**-**	**-**	**-**	**-**	**-**	**6**
**Chen JW, 2014 (55)**	**China**	**2000-2007**	**433**	**209(48.3%)**	**321(74.1%)**	**124/309**	**233/200**	**-**	**-**	**-**	**8**
**Szumilo J, 2009 (56)**	**Poland**	**1995-2001**	**39**	**27(69.2%)**	**36(92.3%)**	**1/38**	**7/32**	**-**	**-**	**-**	**6**
**Lee EJ, 2008 (57)**	**Korea**	**1994-2001**	**251**	**14(6%)**	**110(92%)**	**-**	**-**	**7(3%)**	**-**	**-**	**6**
**Wang Y, 2004 (58)**	**China**	**2000-2000**	**25**	**5(20%)**	**19(76%)**	**9/16**	**15/10**	**-**	**-**	**-**	**7**
**Roh MS, 2004 (59)**	**Korea**	**1996-2003**	**56**	**12(21.4%)**	**51(91.1%)**	**24/32**	**-**	**22(39.3%)**	**27(48.2%)**	**-**	**7**
**Chaves P,1997 (60)**	**Portugal**	**1986-1990**	**37**	**17(45.9%)**	**31(83.8%)**	**8/29**	**-**	**-**	**-**	**-**	**7**
**Sarbia M, 1995 (61)**	**Germany**	**1978-1992**	**161**	**42(26.1%)**	**132(82%)**	**42/119**	**64/97**	**-**	**-**	**-**	**6**
**EAC**											
**Merritt RE, 2020 (62)**	**USA**	**2010-2018**	**215**	**44(20.5%)**	**186(86.5%)**	**55/163**	**85/130**	**53 (24.7%)**	**-**	**OS**	**8**
**Tapias L, 2020 (63)**	**USA**	**2002-2017**	**196**	**104(53.1%)**	**166(84.7%)**	**-**	**61/135**	**-**	**135(68.9%)**	**OS**	**7**
**Turato C, 2019 (64)**	**Italy**	**NR**	**75**	**35(46.7%)**	**67(89.3%)**	**-**	**-**	**-**	**-**	**-**	**6**
**Dislich B, 2017 (65)**	**Switzerland**	**1990-2011**	**112**	**46(41.1%)**	**-**	**43/69**	**-**	**-**	**60(53.6%)**	**-**	**7**
**Drage MG, 2017 (66)**	**USA**	**1989-2011**	**120**	**34(28.3%)**	**97(80.8%)**	**-**	**-**	**46 (38.3%)**	**-**	**-**	**6**
**Patel AK, 2016 (67)**	**USA**	**1996-2015**	**73**	**29(39.7%)**	**67(91.8%)**	**21/37**	**32/38**	**14(19.2%)**	**-**	**-**	**7**
**Thies S, 2016 (68)**	**Germany** **Switzerland**	**1996-2011**	**200**	**63(31.5%)**	**-**	**88/112**	**-**	**107(53.5%)**	**107(53.5%)**	**-**	**7**
**Singhi AD, 2015 (69)**	**USA**	**1997-2009**	**205**	**94(45.9%)**	**170(83%)**	**60/145**	**26/179**	**158(77.1%)**	**-**	**OS**	**8**
**Castonguay MC, 2014 (70)**	**Canada**	**1998-2005**	**103**	**57(55.3%)**	**86(83.5%)**	**38/65**	**35/68**	**-**	**-**	**-**	**6**
**Mehta KS, 2014 (71)**	**USA**	**NR**	**128**	**63(49.2%)**	**95(74.2%)**	**-**	**-**	**-**	**-**	**-**	**6**
**Smith E, 2014 (72)**	**Australia**	**NR**	**65**	**30(46.2%)**	**56(86.2%)**	**25/40**	**28/37**	**-**	**-**	**OS**	**7**
**Lagorce C, 2003 (73)**	**France**	**1976-1997**	**66**	**37(56.1%)**	**63(95.5%)**	**-**	**-**	**-**	**-**	**-**	**6**
**Torres C, 1999 (74)**	**USA**	**1987-1996**	**96**	**31(32.3%)**	**83(86.5%)**	**35/48**	**-**	**-**	**36(37.5%)**	**-**	**6**

LVI, lymphovascular invasion; LNM, Lymph node metastasis; NR, No reported.

### The Significant Correlation Between PNI and Pathological Parameters of EC

Nine, 10, and 6 studies published from 1995 to 2022 were used to analyze the relationship between PNI and gender (6, 21, 23, 30, 46, 49, 53, 55, 75), T stage (6, 21, 23, 30, 46, 49, 53, 55, 61, 75), and N stage (6, 21, 23, 49, 53, 55), respectively.

**Figure 2:**
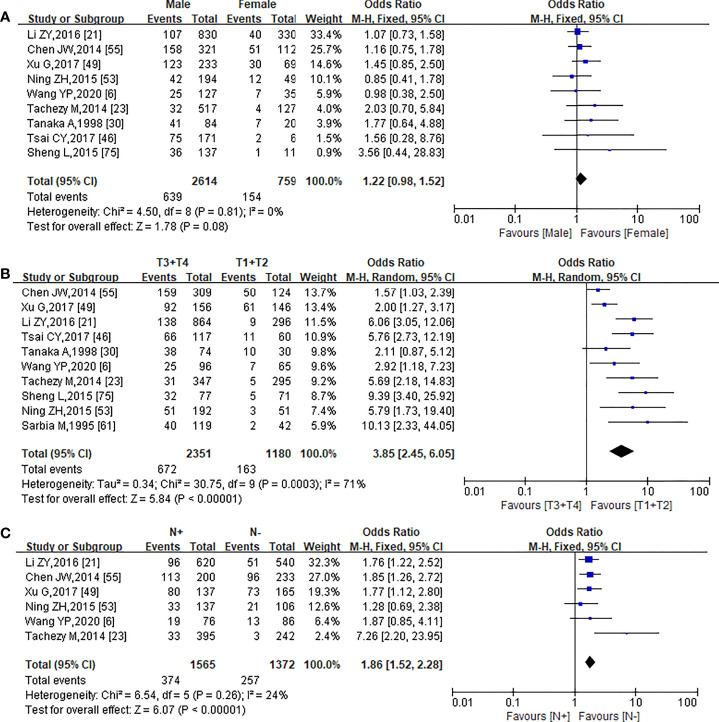
Forest plot of the pooled OR for the association of PNI with Gender **(A)**, pTstage **(B)**, and pN stage **(C)**.

The occurrence of PNI has no correlation with gender (OR = 1.22, 95% CI: 0.98–1.52, *I*² = 0, *p* = 0.08) (**Figure 2A**), while the PNI-positive patients have a significantly higher risk of pT stage (OR = 3.85, 95% CI = 2.45–6.05, *p* < 0.00001) (**Figure 2B**) and pN stage (OR = 1.86, 95% CI = 1.52–2.28, *p* < 0.00001) (**Figure 2C**). In addition, in the meta-analysis for the relationship between PNI and pT stage, no obvious publication bias was observed in the entire funnel plots (**Figure S1B**).

**Figure 3:**
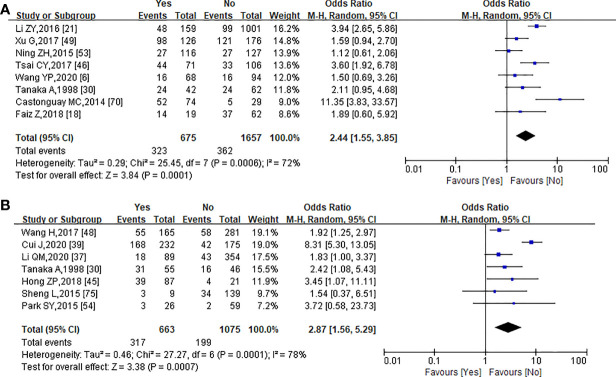
Forest plot analysis of the relationship between PNI and lymphovascularinvasion **(A)**, and lymph node metastasis **(B)**.

A total of 2,332 patients from 8 studies were included in the meta-analysis of the correlation between PNI and LVI (6, 18, 21, 30, 46, 49, 53, 70). In PNI-positive (+) patients, the positive rate of LVI was 47.85% (323/675) and the negative rate of LVI was 52.15% (352/675), while in PNI-negative (−) patients, the positive rate of LVI was 21.85% (362/1,657) and the negative rate of LVI was 78.15% (1,295/1,657). The Chi-square test showed that PNI was significantly correlated with LVI (*χ*^2^ = 156.347, *p* = 0.000, *r* = 0.259). The forest plot was also statistically significant (OR = 2.44, 95% CI = 1.55–3.85, *p* = 0.0001) using a random-effect model for calculation (heterogeneity: *I*² = 72%, *p* = 0.0001) (**Figure 3A**).

Seven studies with a total of 1,738 patients provided data about lymph node metastasis (30, 37, 39, 45, 48, 54, 75). Lymph node metastasis occurred in 317 of the 516 PNI(+) patients (61.43%) and in 346 of the 1,222 PNI(−) patients (28.31%). There was a significant correlation between lymph node metastasis and PNI in EC patients (*χ*^2^ = 168.665, *p* = 0.000, *r* = 0.312). Of these seven studies, there was a significant association between PNI with lymph node metastasis (OR = 2.87, 95% CI = 1.56–5.29, *p* = 0.0007; **Figure 3B**) using a random-effect model for calculation (heterogeneity: *I*² = 85%, *p* = 0.0007).

### The Effect of PNI on 5-Year Overall Survival and 5-Year Disease-Free Survival in EC

**Figure 4:**
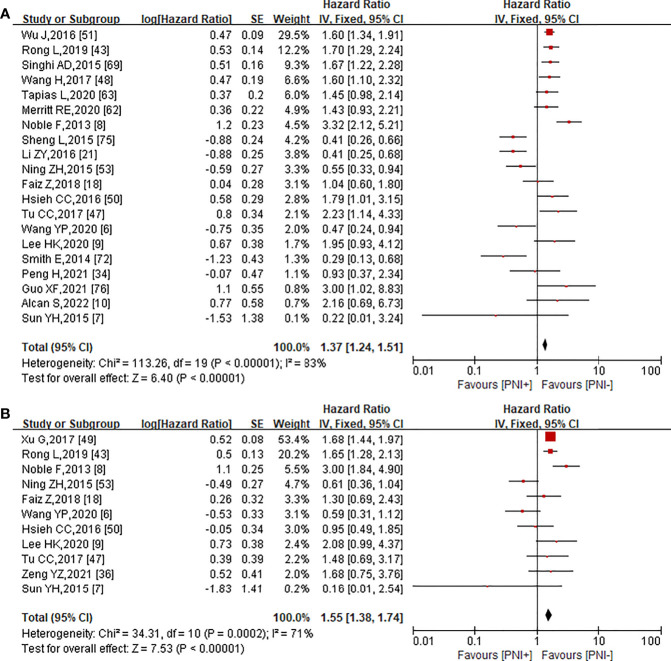
Forest plot analysis for significant correlation between the presence of PNI and5-year OS **(A)**, 5-year DFS **(B)**.

We also studied the effect of PNI on 5-year OS and 5-year DFS of EC patients. HR and 95% CI of OS and DFS were directly reported in 20 (6–10, 18, 21, 34, 43, 47, 48, 50, 51, 53, 62, 63, 69, 72, 75, 76) and 11 (6–9, 18, 36, 43, 47, 49, 50, 53) articles, respectively. Since some studies did not provide HR directly, we used fixed-effect models for the prognostic analysis regardless of the heterogeneity. There was a significant association between PNI and OS (HR = 1.37, 95% CI = 1.24–1.51, *p* < 0.0001; **Figure 4A**) using a fixed-effect model for calculation (heterogeneity: *I*^2^ = 83%, *p* < 0.0001), and the entire funnel plots had obvious publication bias (**Figure S1C**). A meta-analysis of 10 studies on DFS showed that PNI was associated with poor prognosis in EC patients (HR = 1.55, 95% CI = 1.38–1.74, *p* < 0.0001; **Figure 4B**) using a fixed-effect model for calculation (heterogeneity: *I*² = 71%, *p* < 0.0001). In this meta-analysis, a funnel plot was used to assess the publication bias. The entire funnel plots had no obvious publication bias (**Figure S1D**).

## Discussion

PNI was identified in a variety of malignant tumors, such as prostate cancer, pancreatic cancer, and head and neck cancer (2–5). The percentage of patients with PNI in pancreatic ductal adenocarcinoma is 70%–100%, and is closely related to the occurrence of ache (3). In colorectal cancer, PNI is an independent risk factor of recurrence, which indicates a worse phenotype of tumor (3). EC is one of the common malignant tumors with high invasion. PNI often occurs in EC, but there are conflicting reports about the effects on EC of PNI (13, 16, 25, 28). This review and meta-analysis was conducted to better understand the relationship of PNI with the development process and prognosis of EC.

The esophageal nerve includes the vagal nerve and sympathetic nerve. The abundant nerve plexus is mainly distributed in the submucosa and smooth muscle layer and is often accompanied by blood vessels and lymphatic vessels. The development of PNI implies advanced tumor staging, the depth and range of LVI, and lymph node metastasis, as reported by studies (18, 30). However, other studies indicated that there was no relation between PNI and tumor staging, LVI, and lymph node metastasis (6, 54). According to our study, we found that the incidence of PNI in ESCC and EAC was different, but not statistically significant. PNI had a significant association with pT stage, pN stage, LVI, and lymph node metastasis in EC, which are well-known malignant characteristics of EC (77, 78). Moreover, it is worth noting that in cancer tissues with PNI, researchers not only found abundant blood vessels and lymphatic vessels, but also found angiogenesis and lymphangiogenesis, further promoting the development and metastasis of tumor (79, 80). Thus, these results further suggested that PNI was an important feature for the malignant degree of cancer.

It is well known that the malignant degree of EC has a significant association with poor prognosis of EC. Faiz et al. and Noble et al. reported that PNI is positively related to poor prognosis (8, 18), while Li et al. and Dong et al. identified that it was negatively related to poor outcome (21, 22). We evaluated the effect of PNI on 5-year OS and 5-year DFS of EC and found that there was a statically significant association between PNI and OS and DFS. These results indicated that PNI was an independent risk factor for the prognosis of EC. However, no matter what the treatment is, PNI is also significantly associated with worse OS and DFS, and can be evaluated as a prognostic predictor (42, 81).

At present, it is believed that PNI is the result of the interaction between tumor cells and nerves. The occurrence of PNI is not only closely related to the distribution of nerves in tissues and tumor progression, but also associated with the regulation at the molecular level. In ESCC PNI, studies indicated that several genes, such as NF-KB (27), P53 (60), nuclear programmed cell death 4 (PDCD4) (82), and NK1R (83), were significantly positively associated with PNI development, while an inverse correlation was found between platelet counts and PNI (84). The expression of nuclear PDCD4 can predict the prognosis of EC. Moreover, nuclear PDCD4 expression was negatively correlated with PNI (82). Substance P (SP) plays an important role in several types of cancer promotion and progression by binding to its preferential neurokinin 1 receptor (NK1R). NK1R was upregulated, and its overexpression correlated with larger tumor size, deeper tumor invasion, more PNI, and eventually caused poorer OS (83). However, the exact molecular mechanism of PNI in EC remains unclear and is worth further exploring.

In brief, PNI is a dynamic pathological process, and its underlying molecular mechanisms need to be further investigated. Our study only proves that PNI plays an important role in EC. Moreover, our results suggested that PNI can be incorporated into patient stratification factors to make more accurate surgical or treatment plans. This not only greatly improves the survival rate and prognosis of patients, but also enables the further development of precision medicine.

## Conclusion

This review and meta-analysis was conducted to better understand the relationship of PNI with the development process and prognosis of EC. The results indicated that the effect of PNI on poor prognosis is not isolated and associated with gene expression, especially the presence of a number of adverse prognostic factors, such as depth of invasion, clinical stage, LVI, and lymph node metastasis. In general, PNI is a significant indicator of high malignant degree and poor prognosis in EC.

## Data Availability Statement

The original contributions presented in the study are included in the article/**Supplementary Material**, further inquiries can be directed to the corresponding author/s.

## Author Contributions

LB provided the idea and logic of the article, and was responsible for the data collection and statistical processing of it. LY assisted LB to complete the production of pictures and tables, and put forward other ideas. The other authors put forward valuable suggestions and revised and polished the manuscript in the process of writing the article. All authors contributed to the article and approved the submitted version.

## Funding

This work was supported by the National Natural Science Foundation of China (Grant no. 81101686), the Foundation of Henan Educational Committee (Grant no. 21A310030 and Grant no. 22A310024), and the Natural Science Foundation for Young Teachers’ Basic Research of Zhengzhou University (Grant no. JC202035025).

## Conflict of Interest

The authors declare that the research was conducted in the absence of any commercial or financial relationships that could be construed as a potential conflict of interest.

## Publisher’s Note

All claims expressed in this article are solely those of the authors and do not necessarily represent those of their affiliated organizations, or those of the publisher, the editors and the reviewers. Any product that may be evaluated in this article, or claim that may be made by its manufacturer, is not guaranteed or endorsed by the publisher.
