# Supplementary figures and images for: Perineural Invasion Is a Significant Indicator of High Malignant Degree and Poor Prognosis in Esophageal Cancer: A Systematic Review and Meta-Analysis
Source: Front Oncol. 2022 Jun 8;12:816270. doi: 10.3389/fonc.2022.816270 (PMC9213664; doi:10.3389/fonc.2022.816270)

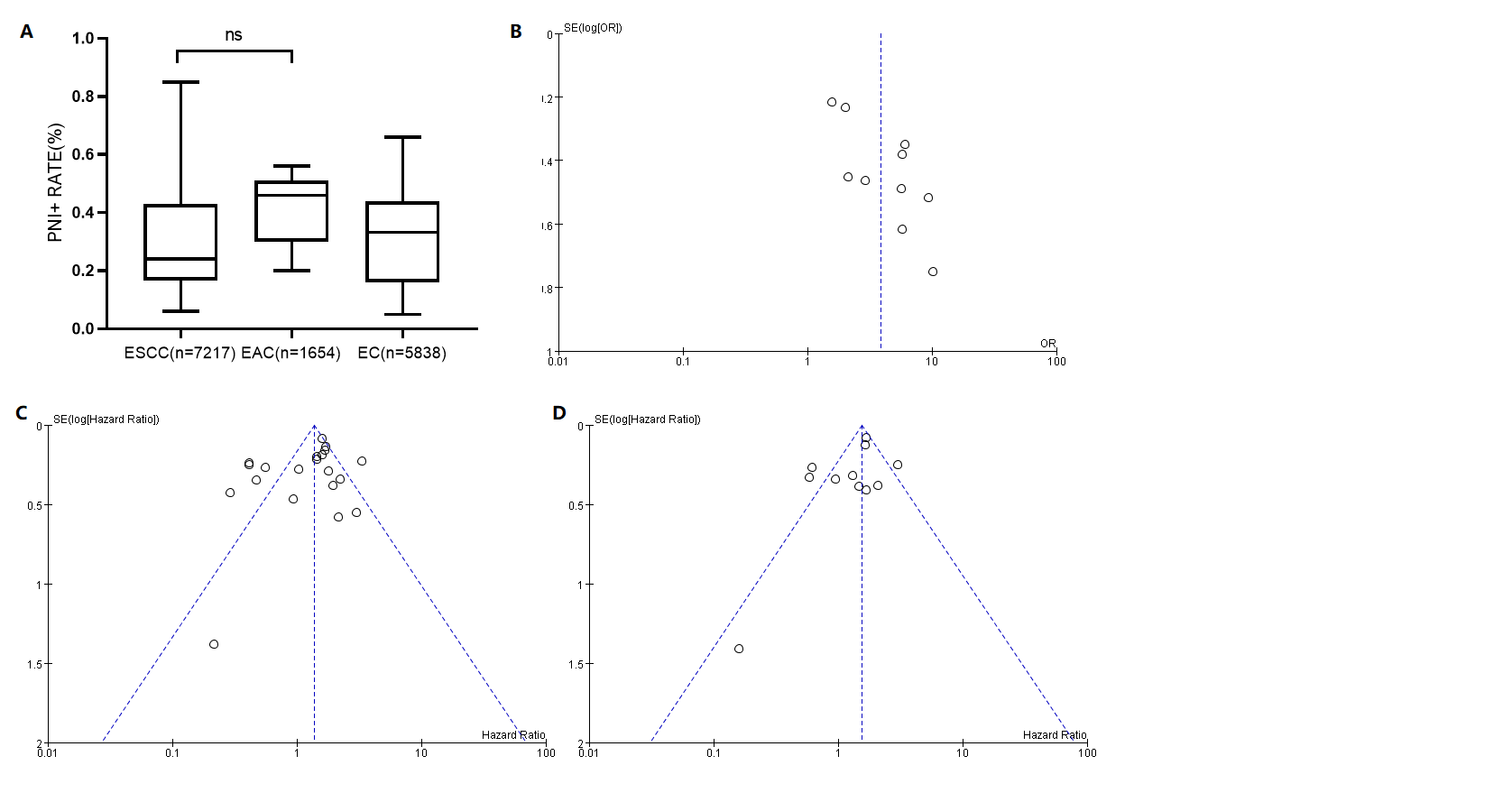

Supplement: Supplementary file 1 [file Image_1.png]
